# S-Scheme BiOCl/MoSe_2_ Heterostructure with Enhanced Photocatalytic Activity for Dyes and Antibiotics Degradation under Sunlight Irradiation

**DOI:** 10.3390/s22093344

**Published:** 2022-04-27

**Authors:** Yan Huang, Fan Chen, Zhipeng Guan, Yusheng Luo, Liang Zhou, Yufeng Lu, Baozhu Tian, Jinlong Zhang

**Affiliations:** 1 Key Laboratory for Advanced Materials and Feringa Nobel Prize Scientist Joint Research Center, Institute of Fine Chemicals, School of Chemistry and Molecular Engineering, East China University of Science and Technology, 130 Meilong Road, Shanghai 200237, China; huangyan_135@126.com (Y.H.); avazaza0131@icloud.com (F.C.); guan10130725@126.com (Z.G.); lyslouis217@126.com (Y.L.); luyufenghd@163.com (Y.L.); 2 Research Institute of Physical and Chemical Engineering of Nuclear Industry, 168 Jintang Road, Tianjin 300180, China; 3 State Environmental Protection Key Lab of Environmental Risk Assessment and Control on Chemical Processes, School of Resources & Environmental Engineering, East China University of Science and Technology, 130 Meilong Road, Shanghai 200237, China; zhouliang@ecust.edu.cn; 4 Key Laboratory of Specially Functional Polymeric Materials and Related Technology (Ministry of Education), East China University of Science and Technology, 130 Meilong Road, Shanghai 200237, China

**Keywords:** photocatalysis, dye, antibiotics, S-scheme heterojunction, photocatalytic activity, reactive species, organic pollutant

## Abstract

Semiconductor photocatalysis is considered to be a promising technique to completely eliminate the organic pollutants in wastewater. Recently, S-scheme heterojunction photocatalysts have received much attention due to their high solar efficiency, superior transfer efficiency of charge carriers, and strong redox ability. Herein, we fabricated an S-scheme heterostructure BiOCl/MoSe_2_ by loading MoSe_2_ nanosheets on the surface of BiOCl microcrystals, using a solvothermal method. The microstructures, light absorption, and photoelectrochemical performances of the samples were characterized by the means of SEM, TEM, XRD, transient photocurrents, electrochemical impedance, and photoluminescence (PL) spectra. The photocatalytic activities of BiOCl, MoSe_2_, and the BiOCl/MoSe_2_ samples with different MoSe_2_ contents were evaluated by the degradation of methyl orange (MO) and antibiotic sulfadiazine (SD) under simulated sunlight irradiation. It was found that BiOCl/MoSe_2_ displayed an evidently enhanced photocatalytic activity compared to single BiOCl and MoSe_2_, and 30 wt.% was an optimal loading amount for obtaining the highest photocatalytic activity. On the basis of radical trapping experiments and energy level analyses, it was deduced that BiOCl/MoSe_2_ follows an S-scheme charge transfer pathway and •O_2_^−^, •OH, and h^+^ all take part in the degradation of organic pollutants.

## 1. Introduction

With the rapid development of urbanization and industrialization, water pollution has become more and more serious and imparted huge adverse effects on aquatic ecosystems, human health, and the development of economy and society [1,2,3]. In recent decades, the removal of noxious organic pollutants in wastewater, such as drugs [4,5], dyes [6,7], and antibiotics [8], has become a big challenge that must be managed. For instance, the antibiotics always bring about side effects on ecosystems and human health by inducing the proliferation of bacterial drug resistance [6]. The carcinogenic and teratogenic dyes can enter the human body along with the polluted water, leading to the appearance of cancers and other serious illnesses. To eliminate these organic pollutants, a series of techniques, such as physical adsorption, micro-biological degradation, and chemical oxidation, have been applied in the remediation of organic pollutants [1,9]. However, these strategies are still insufficient to completely remove the water-borne organic pollutants because of their low efficiency, as well as the formation of secondary waste products [10,11,12]. Alternatively, semiconductor photocatalysis has received much attention as a promising solution to completely eliminate the organic contaminants in wastewater [1,2,3,4,5,6,7,13,14,15,16,17,18,19]. As a semiconductor with wide bandgap, BiOCl is considered to be an ideal photocatalyst for the decomposition of organic pollutants in wastewater under UV light [20,21]. The main weakness of BiOCl is that it cannot respond to visible light, severely blocking its application in the whole solar spectrum. To solve this problem, the researchers have developed many strategies, such as fabricating oxygen vacancies, depositing with metals, constructing heterojunctions, and so on [22,23,24,25,26,27,28,29,30]. Although these approaches can extend the light response of BiOCl to the visible region, they inevitably decrease the redox ability of the photogenerated electrons and holes. In this regard, the researchers further exploited a series of all-solid-state and direct Z-scheme composite semiconductors to avoid decreasing the redox ability of photogenerated charge carriers [13,14,31,32,33,34,35,36,37,38]. Recently, Yu et al. proposed a novel S-scheme heterojunction theory and reasonably explained the transfer pathway of photogenerated charge carriers in the two semiconductors [39]. From then on, a series of S-scheme photocatalytic materials have been reported and successfully applied in the fields of environment and energy [40,41,42,43,44,45,46,47].

Layer-structured molybdenum selenide (MoSe_2_) has a narrow band gap (about 1.3–1.9 eV) [48,49], which means it can respond to the whole UV-visible-near-infrared (UV-Vis-NIR) light. However, its multilayer structure and narrow bandgap usually lead to the high recombination rate of photogenerated charge carriers [50]. In this regard, coupling MoSe_2_ with other semiconductors with wide bandgaps is an ideal strategy to take its advantages and simultaneously avoid its flaws. So far, several composite MoSe_2_-based photocatalysts have been exploited [51,52,53,54]. However, to the best our knowledge, the S-scheme heterojunction photocatalyst based on MoSe_2_ and BiOCl has never been studied.

Herein, we first constructed the S-scheme heterojunction BiOCl/MoSe_2_ photocatalyst by loading MoSe_2_ nanosheets on the surface of BiOCl microcrystals, using a solvothermal method. The morphology and crystalline structures of the as-prepared samples were characterized by the means of scanning electron microscopy (SEM), transmission electron microscopy (TEM), and high-resolution transmission electron microscopy (HR-TEM). The light absorption properties of the samples were analyzed by UV-Vis diffuse reflectance spectroscope (DRS). The photoelectric properties and the separation rate of charge carriers were investigated using transient photocurrents, electrochemical impedance, and photoluminescent (PL) spectra. The photocatalytic activities of BiOCl and the different BiOCl/MoSe_2_ samples were evaluated by the degradation of azo dye methyl orange (MO) and antibiotic sulfadiazine (SD) under simulated sunlight irradiation. On the basis of the radical trapping experiments and potential analyses of BiOCl and MoSe_2_ conduction bands (CB) and valence bands (VB), the possible photocatalytic mechanism of S-scheme BiOCl/MoSe_2_ was proposed.

## 2. Materials and Methods

### 2.1. Materials

Bismuth nitrate pentahydrate (Bi(NO_3_)_3_·5H_2_O) and absolute ethanol (C_2_H_5_OH) were provided by Sinopharm Chemical Reagent Co., Ltd., Shanghai, China. Selenium powder, sodium molybdate dihydrate (Na_2_MoO_4_·2H_2_O), and sodium borohydride (NaBH_4_) were purchased from Shanghai Adamas Reagent Co., Ltd., Shanghai, China. Potassium chloride (KCl) was obtained from Shanghai Lingfeng Chemical Reagent Co., Ltd., Shanghai, China. All the reagents were analytically pure grade and used as received without further purification. Milli-Q water was homemade and the resistivity was 18.2 MΩ cm.

### 2.2. Synthesis of BiOCl/MoSe_2_

BiOCl nanosheets were prepared using a hydrothermal method, similar to the previous report [55]. The detailed procedures were as follows: Firstly, 1 mmol Bi(NO_3_)_3_·5H_2_O and 1 mmol KCl were successively dispersed in 15 mL deionized water and stirred at room temperature for 1 h. Then, the mixture was transferred into a 50 mL Teflon-lined stainless-steel autoclave and placed in an oven to react at 160 °C for 24 h. Subsequently, the suspension was cooled to room temperature and the precipitation was washed, respectively, with deionized water and ethanol three times. Finally, the product was dried in a vacuum drying oven at 70 °C for 8 h, denoted as BiOCl.

BiOCl/MoSe_2_ was synthesized via a modified solvothermal method [56]: Firstly, 200.5 mg BiOCl, 0.079 mmol Na_2_MoO_4_·2H_2_O, 0.158 mmol selenium powder, and 0.079 mmol NaBH_4_ were added into a 25 mL mixture solution of ethanol and water with a volume ratio of 1:1. After the mixture was stirred at room temperature for 1 h, the obtained homogeneous mixture was transferred into a 50 mL Teflon-lined stainless-steel autoclave and kept at 180 °C for 12 h. Then, the autoclave was cooled to room temperature and the obtained precipitate was washed with deionized water and ethanol three times, respectively. Finally, the obtained product was dried in a vacuum drying oven at 70 °C for 8 h. The theoretical loading amount of the MoSe_2_ sample was 10 wt.%, denoted as BiOCl/MoSe_2_-10. By changing the dosages of Na_2_MoO_4_·2H_2_O, selenium powder, and NaBH_4_, the BiOCl/MoSe_2_ samples with 30 wt.% and 50 wt.% MoSe_2_ contents were also synthesized, denoted as BiOCl/MoSe_2_-30 and BiOCl/MoSe_2_-50, respectively. Pure MoSe_2_ was further prepared by the same method, except that BiOCl was not added.

### 2.3. Characterization

The morphologies of the obtained samples were observed via scanning electron microscope (SEM, TESCAN VEGA 3 SBH), transmission electron microscope (TEM, JEM2000EX), and high-resolution transmission electron microscope (HR-TEM, JEOJ JEM2100). The crystalline structures of the samples were analyzed using a Riguku D/Max 2550 VB/PC X-ray diffractometer with Cu Kα (λ = 1.5406 A) radiation, operated at a voltage of 40 kV and a current of 40 mA. The UV-Vis diffuse reflectance spectra of the samples were recorded on a SHIMADZU UV-2450 spectrophotometer and a Lambda 950 spectrophotometer, equipped with an integrating sphere assembly, using BaSO_4_ as the reference material. The photoluminescence (PL) spectra were tested on a Shimadzu RF5301PC fluorescence spectrophotometer and the 320 nm line of Xe lamp was used as the excitation source. The transient photocurrents, electrochemical impedance, and Mott–Schottky spectra were measured by a Zahner electrochemical workstation equipped with a three-electrode system, in which the platinum electrode and saturated calomel electrode were used as the counter electrode and reference electrode, respectively, and 0.2 mg photocatalyst sample was coated on 1.5 cm^2^ FTO glass as the working electrode. The transient photocurrent and Mott–Schottky tests were performed in a 0.5 M Na_2_SO_4_ aqueous solution and a 300 W Xe lamp with AM 1.5 filter as the light source. A mixed aqueous solution of 2.0 mM K_3_[Fe(CN)_6_], 2.0 mM K_4_[Fe(CN)_6_], and 0.5 M KCl was used as the electrolyte for the electrochemical impedance tests.

### 2.4. Photocatalytic Activity Measurement

The photocatalytic activities of the prepared samples were evaluated by the degradation of methyl orange (MO) and sulfadiazine (SD) under simulated sunlight irradiation, using a 300 W Xe lamp with AM1.5 as the light source. For each measurement, a 50 mg photocatalyst was dispersed in a 50 mL MO/or SD (20 mg/L) solution in a quartz tube and stirred in the dark for 30 min to achieve the adsorption–desorption of MO/or SD on the surface of the photocatalyst. At a given time interval, 5 mL of the mixture solution was withdrawn, centrifuged, and filtered to remove the remaining particles. The residual concentrations of MO and SD were determined using a UV-Vis spectrophotometer and a high-performance liquid chromatograph, respectively.

## 3. Results and Discussion

### 3.1. Morphological and Crystalline Structures

**Figure 1:**
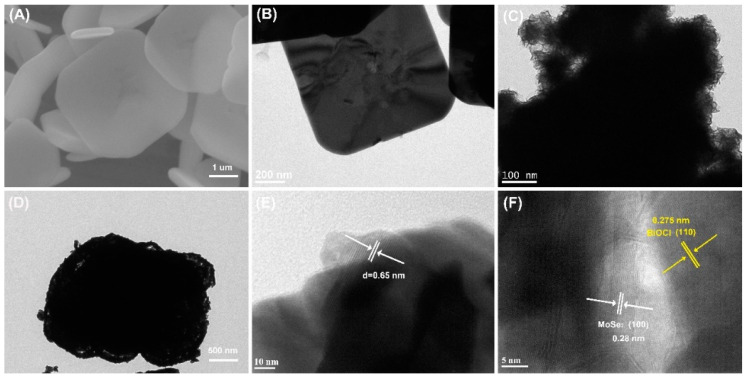
(**A**) SEM image of BiOCl. (**B**–**D**) TEM images of (**B**) BiOCl, (**C**) MoSe_2_, and (**D**) BiOCl/MoSe_2_-30. (**E**,**F**) HR-TEM images of BiOCl/MoSe_2_-30.

The morphological structures of the samples were observed by SEM, TEM, and HR-TEM images. As shown in Figure 1A,B, the surface of BiOCl sheets seems to be smooth and the width and thickness of BiOCl sheets are in the range of 1−4.5 μm and 300−400 nm, respectively. From the TEM images of BiOCl and BiOCl/MoSe_2_-30, it can be seen that the block-structured MoSe_2_ consists of many thin nanosheets (Figure 1C), which are uniformly wrapped on the surface of BiOCl sheets to form a shell structure (Figure 1D). The lattice structure of BiOCl/MoSe_2_-30 was further analyzed using HR-TEM images. In Figure 1E, the lattice spacing was measured to be 0.65 nm, attributed to the (0 0 2) crystal planes of 2H phase MoSe_2_ [57]. In Figure 1F, the lattice spacing of 0.275 nm corresponds to BiOCl (1 1 0) crystal planes, while that of 0.28 nm is attributed to MoSe_2_ (1 0 0) crystal planes. These results demonstrate the formation of a BiOCl/MoSe_2_ heterojunction structure [58].

**Figure 2:**
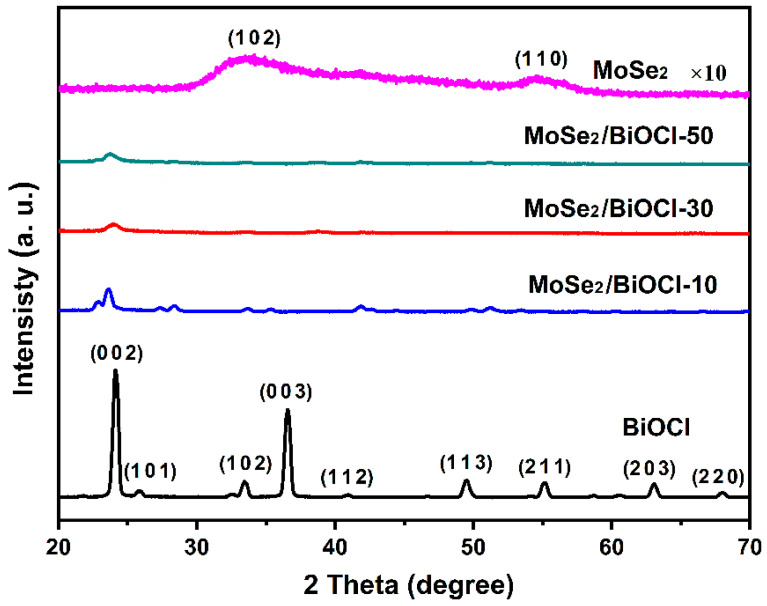
XRD patterns of MoSe_2_, BiOCl, BiOCl/MoSe_2_-10, BiOCl/MoSe_2_-30, and BiOCl/MoSe_2_-50.

The crystalline structures of the synthesized samples were analyzed by X-ray diffraction patterns (XRD). As shown in Figure 2, BiOCl presents the diffraction peaks at 2θ = 24.1°, 25.9°, 33.4°, 36.5°, 40.9°, 49.7°, 54.1°, 63.1°, and 68.1°, attributed to BiOCl (0 0 2), (1 0 1), (1 0 2), (0 0 3), (1 1 2), (1 1 3), (2 1 1), (2 0 3), and (2 2 0) crystal planes, respectively (JCPDS No. 06-0249) [54]. In contrast, BiOCl/MoSe_2_-10, BiOCl/MoSe_2_-30, and BiOCl/MoSe_2_-50 exhibit an obvious diffraction peak at 24.1°, while the other characteristic peaks become very weak, due to the resistance of the thick MoSe_2_ shell layer to X-ray. Even enlarged 10 times in intensity, the diffraction peaks of MoSe_2_ (1 0 2) and (1 1 0) are still very weak and broad, which is probably ascribable to both its low crystallinity as well as the very thin sheet structure.

### 3.2. Light Absorption and PL Properties

**Figure 3:**
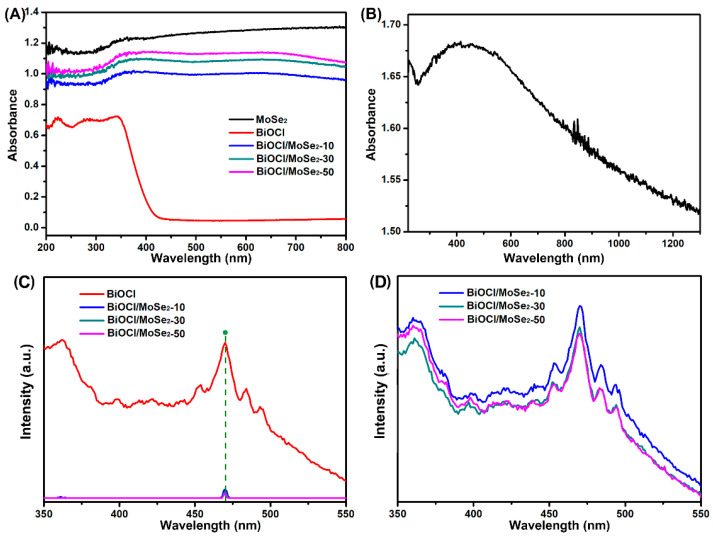
(**A**) UV−Vis DRS spectra of MoSe_2_, BiOCl, BiOCl/MoSe_2_-10, BiOCl/MoSe_2_-30, and BiOCl/MoSe_2_-50 in the UV and visible light region. (**B**) DRS spectra of MoSe_2_ in the UV, visible light, and NIR region. (**C**) PL spectra of BiOCl, BiOCl/MoSe_2_-10, BiOCl/MoSe_2_-30, and BiOCl/MoSe_2_-50. (**D**) Enhanced PL spectra of BiOCl/MoSe_2_-10, BiOCl/MoSe_2_-30, and BiOCl/MoSe_2_-50.

The optical properties of MoSe_2_, BiOCl, and BiOCl/MoSe_2_ were investigated by UV-Vis DRS and PL spectra. As shown in Figure 3A,B, pure BiOCl only can absorb UV light, while MoSe_2_ displays strong light absorption in the whole UV-Vis-NIR region. After coupling with MoSe_2_, all the BiOCl/MoSe_2_ samples exhibit a significantly enhanced absorption in the visible and NIR region, and the absorption intensity gradually increases with the increase of MoSe_2_ content. PL spectrum is a useful technique to investigate the trapping, migration, and transfer efficiency of the photogenerated charge carriers in semiconductor photocatalysts [31,59,60]. Herein, we tested the PL spectra of BiOCl and the different BiOCl/MoSe_2_ samples at room temperature with an excitation wavelength of 320 nm. As displayed in Figure 3C, BiOCl exhibits a strong PL emission band in the range of 350–550 nm, while all the BiOCl/MoSe_2_ samples only have a very weak PL emission peak at 470 nm. After increasing the luminous flux of excitation light, the three BiOCl/MoSe_2_ samples also exhibit the PL emission bands in the range of 350–550 nm, similar to that of BiOCl (Figure 3D). The PL intensity of BiOCl/MoSe_2_-30 is near to that of BiOCl/MoSe_2_-50 and obviously weaker than that of BiOCl/MoSe_2_-10. These results indicate that the coupling of BiOCl and MoSe_2_ can effectively restrain the recombination of photogenerated charge carriers and that 30 wt.% is the optimal MoSe_2_ loading amount for effectively separating the photogenerated electrons and holes.

### 3.3. Photoelectric Characteristics

**Figure 4:**
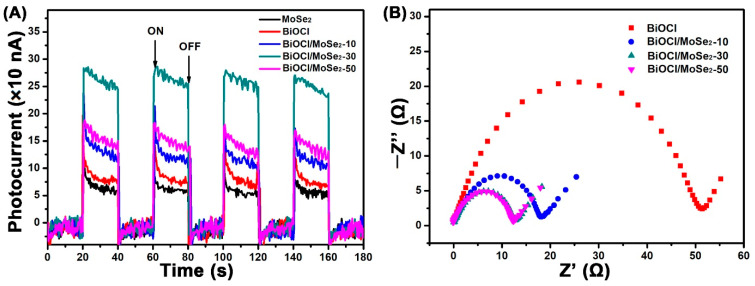
(**A**) Transient photocurrents of MoSe_2_, BiOCl, BiOCl/MoSe_2_-10, BiOCl/MoSe_2_-30, and BiOCl/MoSe_2_-50 under simulated sunlight irradiation; (**B**) Nyquist plots of the electrochemical impedance spectra of BiOCl, BiOCl/MoSe_2_-10, BiOCl/MoSe_2_-30, and BiOCl/MoSe_2_-50.

The photoelectric characteristics of BiOCl and the different BiOCl/MoSe_2_ samples were investigated by transient photocurrent measurements, which can further disclose the production, separation, and transfer efficiency of photogenerated charge carriers in these samples. As shown in Figure 4A, both BiOCl and MoSe_2_ exhibit very weak photocurrent intensity due to the low sunlight response ability and the high recombination rate of photo-generated electrons and holes, respectively. In contrast, all the BiOCl/MoSe_2_ composite photocatalysts display obviously enhanced current photocurrent intensity, indicating that the formation of a heterojunction structure can effectively promote the separation and transfer of photogenerated charge carriers. Amongst these samples, BiOCl/MoSe_2_-30 shows the highest photocurrent intensity, which is about four times that of pure BiOCl. For BiOCl/MoSe_2_-50, its photocurrent intensity is evidently weaker than that of BiOCl/MoSe_2_-30, resulting from the shielding of excess MoSe_2_ to light [61]. The electrochemical impedance spectra (EIS) can be used to disclose the dynamics of the mobile and bound charges in the interfacial or bulk regions of semiconductors, and the smaller curvature radius usually implies the weaker resistance to charge transfer [14,62,63]. In the EIS Nyquist spectra of Figure 4B, all the BiOCl/MoSe_2_ samples exhibit much smaller semicircle diameters than BiOCl, implying that coupling MoSe_2_ can effectively decrease the transfer resistance of the carriers in BiOCl. As the loading amount of MoSe_2_ increases from 10 wt.% to 30 wt.%, the semicircle diameter of the EIS curve obviously becomes smaller and it almost has no change when the loading amount of MoSe_2_ is further increased to 30 wt.%. Combining the results of the transient photocurrents and EIS spectra, it can be concluded that 30 wt.% is the optimal MoSe_2_ loading amount for effectively facilitating the production, separation, and transfer of photogenerated change carriers.

### 3.4. Photocatalytic Activity and Mechanism

**Figure 5:**
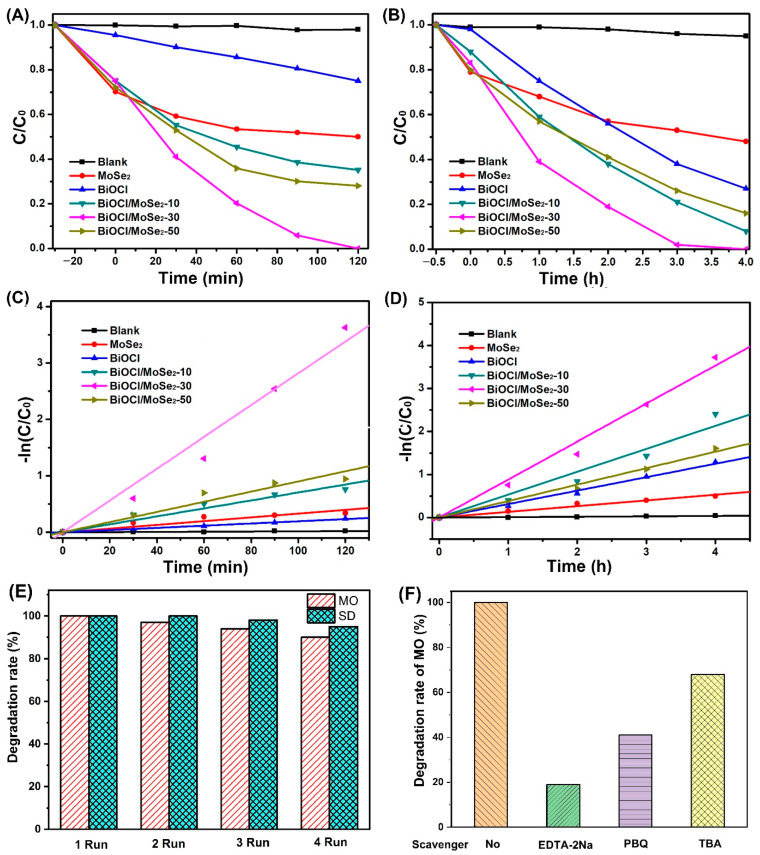
(**A**,**B**) Photocatalytic degradation curves of (**A**) MO and (**B**) SD over the different photocatalysts under simulated sunlight irradiation. Corresponding fitted degradation kinetic curves of (**C**) MO and (**D**) SD. (**E**) Cyclic photocatalytic degradations of MO and SD over BiOCl/MoSe_2_-30. The reaction time of each cycle experiment for MO is 120 min and that for SD is 4 h. (**F**) Photocatalytic degradation rates of MO over BiOCl/MoSe_2_-30 in the presence of different radical scavengers.

Figure 5A,B presents the degradation curves of MO and SD over the different photocatalysts under simulated sunlight irradiation, respectively. In the absence of photocatalyst, the concentrations of MO and SD almost have no change under simulated sunlight irradiation, indicating that they have high photostability. Both pure BiOCl and MoSe_2_ exhibit low photocatalytic activity for MO and SD degradation, which is because BiOCl cannot respond to visible light while MoSe_2_ has the high recombination rate of photogenerated electrons and holes. Compared to pure MoSe_2_ and BiOCl, all the BiOCl/MoSe_2_ samples show evidently enhanced photocatalytic activity for MO and SD degradation, because the heterojunction structure between MoSe_2_ and BiOCl can effectively restrain the recombination of photogenerated electrons and holes. To more accurately compare the photocatalytic activities of BiOCl and the different BiOCl/MoSe_2_ samples, we further fitted the kinetic curves of MO and SD degradations over these samples. From Figure 5C,D, it can be seen that the degradations of MO and SD over these photocatalysts follow the pseudo first-order kinetic reaction. By comparing the reaction kinetic constants in Table 1, we know that BiOCl/MoSe_2_-30 possesses the highest photocatalytic activity among all the samples.

Given that photostability is very important to a photocatalyst for its practical applications, we further tested the photostability of BiOCl/MoSe_2_-30 using the cyclic degradation experiments of MO and SD under simulated sunlight irradiation. As shown in Figure 5E, the degradation rates of MO and SD only display a slight decrease after four cycles, probably due to the inevitable loss of photocatalysts during the recycle runs. This result indicates that BiOCl/MoSe_2_-30 is a stable photocatalyst under simulated sunlight irradiation. In the photocatalytic degradation process, the reactive species that take part in the organic pollutant decomposition mainly include hydroxyl radical (•OH), superoxide radical (•O_2_^−^), and hole (h^+^). Herein, we identified the produced reactive species over BiOCl/MoSe_2_-30 in the organic decomposition process by addition of radical trapping agents. It is known that •OH, h^+^, and •O_2_^−^ can be quenched by tert-butanol (TBA), EDTA-2Na, and p-benzoquinone (PBQ), respectively. As shown in Figure 5F, the degradation rate of MO was evidently inhibited after addition of EDTA-2Na, PBQ, and TBA, implying that all h^+^, •O_2_^−^, and •OH take part in the degradation of MO. The effect of these species for MO degradation deceases in the order of h^+^ > •O_2_^−^ > •OH.

To clarify the migration pathways of photogenerated charge carriers in BiOCl/MoSe_2_, it is necessary to identify the conduction band (CB) and valence band (VB) potentials of MoSe_2_ and BiOCl. In our previous studies [14,62], we have calculated the potentials of BiOCl CB and VB, which are +0.14 eV and +3.51 eV, respectively. Herein, we estimated the potentials of MoSe_2_ CB and VB by analyzing its UV-Vis absorption spectrum and Mott–Schottky curve.

**Figure 6:**
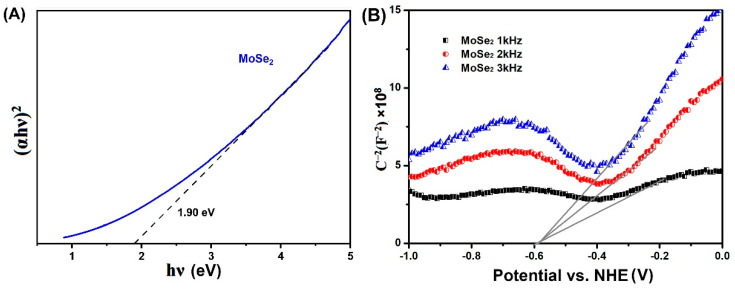
(**A**) Plots of (αhν)1/2 (MoSe2) versus photon energy (hν); (**B**) Mott–Schottky plots of MoSe_2_.

Firstly, the bandgap energy of MoSe_2_ nanosheets was calculated using Tauc plot via the following Kubelka–Munk equation [64]:

(αhν)^2^ = A(hν − E_g_) (1)

where h, α, ν, A, and E_g_ are the Planck constant, absorption coefficient, light frequency, constant value, and bandgap energy, respectively. As shown in Figure 6A, the bandgap energy of MoSe_2_ was estimated to be 1.9 eV, similar to the value of the previous reports [52,65,66]. Then, the potential of MoSe_2_ CB edge was determined by Mott–Schottky analysis [67]. As shown in Figure 6B, the potential of MoSe_2_ CB (E_CB_) was estimated using the extrapolation of the Mott–Schottky plots at different frequencies (1 kHz, 2 kHz, and 3 kHz) to be −0.59 V (vs. NHE). According to the equation of E_VB_ = E_CB_ + E_g_ (E_VB_ is the potential of VB), the potential of MoSe_2_ VB was further calculated to be 1.31 eV.

On the basis of the CB and VB potentials of BiOCl and MoSe_2_, BiOCl/MoSe_2_ should be ascribed to one of the three types of heterojunction, i.e., Type-II, direct Z-scheme, and S-scheme. Firstly, assuming that BiOCl/MoSe_2_ is a Type-II semiconductor, the electrons on MoSe_2_ CB would migrate to BiOCl CB. Given that the potential of BiOCl CB (0.14 eV vs. NHE) is more positive than E_0_(O_2_/•O_2_^−^) (−0.33 eV vs. MHE) [68,69,70], the adsorbed O_2_ cannot be reduced by the electrons on BiOCl CB to form •O_2_^−^**.** Similarly, since the potential of MoSe_2_ VB (1.31 eV vs. NHE) is more negative than E_0_(•OH/OH^−^) (1.99 eV vs. NHE) [68,69,70], the holes on MoSe_2_ VB cannot oxidize OH^–^ into •OH. However, the presence of •O_2_^−^ and •OH has been proved by the radical trapping experiments (Figure 5F), implying that BiOCl/MoSe_2_ is not a traditional Type-II semiconductor and the electrons for •O_2_^−^ production and the holes for •OH production come from the MoSe_2_ CB and BiOCl VB, respectively. Moreover, Z-scheme heterojunction also has a theoretical problem in explaining the transfer pathway of photogenerated electrons and holes in BiOCl/MoSe_2_: from the perspective of charge transfer, the electrons on MoSe_2_ CB will preferentially recombine with the holes on BiOCl VB, rather than the electrons on BiOCl CB recombine with the holes on MoSe_2_ VB.

The S-scheme heterojunction is more reasonable to illustrate the transfer pathway of photogenerated electrons and holes in BiOCl/MoSe_2_ [39,41,71,72]—in this composite photocatalytic system, BiOCl is the oxidation photocatalyst (OP) and MoSe_2_ is the reduction photocatalyst (RP), both of which form an S-scheme heterojunction [39,41,71,72]. After the two components are in close contact, the electrons in MoSe_2_ spontaneously transfer to BiOCl, producing an electron depletion layer and electron accumulation layer near the interface of MoSe_2_ and BiOCl, respectively. Thus, MoSe_2_ would be positively charged and BiOCl would be positively charged, forming an internal electric field directing from MoSe_2_ to BiOCl. Meanwhile, after BiOCl and MoSe_2_ contact together, their Fermi energy should be aligned to the same level. Thus, the Fermi levels of BiOCl and should upward shift and upward shift, respectively, together with the band bending at their interfaces. Both the coulomb force of electric field and the band bending urge the photogenerated electrons from BiOCl to recombine with the holes from MoSe_2_ VB. Due to the band bending, the electrons on MoSe_2_ CB and holes on BiOCl will be reserved.

**Figure 7:**
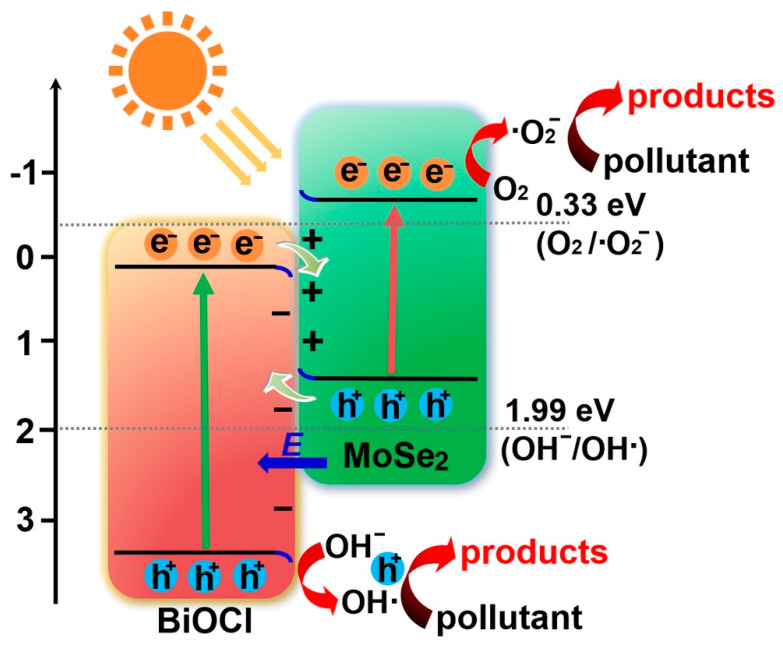
Proposed photocatalytic mechanism of S-scheme BiOCl/MoSe_2_.

Based on the above experimental results and analyses, the degradation mechanism of organic pollutants over S-scheme BiOCl/MoSe_2_ was proposed: As illustrated in Figure 7, under simulated sunlight irradiation, both BiOCl and MoSe_2_ can produce holes on their VB and electrons on their CB. Using the acceleration of internal electric field, the photogenerated electrons on BiOCl CB and the holes on MoSe_2_ would be recombined. As a result, the powerful electrons on MoSe_2_ CB and the powerful holes on BiOCl VB would be reserved. Subsequently, the electrons on MoSe_2_ CB would react with adsorbed O_2_ to form •O_2_^−^. Meanwhile, some holes on the BiOCl VB would oxidize OH^−^ to produce •OH. All of •O_2_^−^, •OH, and h^+^ take part in the degradation of organic pollutants.

## 4. Conclusions

In summary, S-scheme BiOCl/MoSe_2_ heterojunction was fabricated via a modified solvothermal method. It was found that the thin MoSe_2_ nanosheets are uniformly wrapped on the surface of BiOCl microcrystals to form a shell structure. The MoSe_2_ diffraction peaks of MoSe_2_ and the different BiOCl/MoSe_2_ samples are very weak due to its low crystallinity and thin layer structure. The UV-Vis diffuse reflectance spectra show that all the BiOCl/MoSe_2_ samples exhibit a significantly enhanced absorption in the visible and near-infrared light region when compared with BiOCl, and the absorption intensity gradually increases with the increase of MoSe_2_ content. From the photoluminescence spectra, transient photocurrents, and electrochemical impedance spectra, it can be concluded that the BiOCl/MoSe_2_ heterojunction can effectively promote the transfer of photogenerated charge carriers. The results of MO and SD degradations indicate that all the BiOCl/MoSe_2_ samples display an evidently enhanced photocatalytic activity compared to single BiOCl and MoSe_2_, and the optimal MoSe_2_ loading amount for obtaining the highest photocatalytic activity is 30 wt.%. The radical trapping experiments disclosed that all h^+^, •O_2_^−^, and •OH take part in the degradation of organic pollutants and h^+^ plays a more important role than •O_2_^−^ and •OH. By further analyzing the potentials of BiOCl and MoSe_2_ CB and VB, it can be deduced that the BiOCl/MoSe_2_ follows an S-scheme photocatalytic mechanism. We think that this study provides a reference for fabricating the S-scheme photocatalytic materials to eliminate the organic pollutants in wastewater under sunlight irradiation.

## Figures and Tables

**Table 1:** The kinetic constants of photocatalytic degradation of MO and SD over the different samples.

Sample	MoSe_2_	BiOCl	BiOCl/MoSe_2_-10	BiOCl/MoSe_2_-30	BiOCl/MoSe_2_-50
MO (min^−1^)	0.0020	0.0027	0.0063	0.0307	0.0082
SD (h^−1^)	0.1246	0.3258	0.5829	0.9323	0.4004
